# Postoperative Pain After Endodontic Treatment in HIV‐Positive Patients Under HAART: A Prospective Observational Cohort Study

**DOI:** 10.1111/iej.70110

**Published:** 2026-02-04

**Authors:** Marcos Felipe Iparraguirre Nuñovero, Marco Antonio Hungaro Duarte, Luciana Reis Azevedo Alanis, Bruno Cavalini Cavenago, Ulisses Xavier da Silva Neto, Everdan Carneiro

**Affiliations:** ^1^ Pontifícia Universidade Católica do Paraná (PUCPR) Curitiba Brazil; ^2^ University of São Paulo Bauru Brazil; ^3^ Federal University of Paraná (UFPR) Curitiba Brazil

## Abstract

**Aim:**

To compare post‐operative pain following root canal treatment (RCT) in HIV‐positive patients receiving highly active antiretroviral therapy (HAART) and HIV‐negative patients and to assess the influence of systemic condition on endodontic pain.

**Methodology:**

This investigation was designed as a prospective observational cohort study, following the PROBE 2023 guidance. A total of 60 patients (30 HIV‐positive under HAART for at least three years with undetectable viral load, and 30 HIV‐negative) with pulp necrosis and asymptomatic apical periodontitis were included. All teeth were treated in a single visit by an endodontic specialist using standardised chemo‐mechanical preparation with reciprocating instruments, irrigation with 2.5% sodium hypochlorite and 17% EDTA, and single‐cone obturation with AH Plus sealer. Patients without preoperative pain (VAS = 0) were included. Post‐operative pain was assessed using a VAS, 0–10 at 6, 12, 24, 48 and 72 h and 7 days post‐treatment. Statistical analysis was performed using the chi‐square test, Kolmogorov–Smirnov test, and Mann–Whitney *U* test (95% confidence level).

**Results:**

No significant differences were found between groups regarding sex, age or tooth location (*p* > 0.05). Both groups reported postoperative pain in the first 24 h, with no significant differences (*p* > 0.05). At 48 h, however, all HIV‐positive patients reported complete pain relief, whereas the HIV‐negative group still presented residual pain, showing a statistically significant difference (*p* = 0.021). At 72 h and 7 days, both groups reported no pain (VAS = 0). Overall, postoperative pain prevalence exceeded 45% in the first 24 h, regardless of systemic condition.

**Conclusions:**

Postoperative pain patterns were similar in HIV‐positive patients under HAART and HIV‐negative individuals, except for earlier pain resolution in the HIV‐positive group at 48 h. These findings suggest that the systemic condition does not negatively affect the short‐term management of endodontic pain.

## 1 Introduction

Since the introduction of HAART in 1996, a significant transformation has occurred in the management of HIV infection. Studies have shown a remarkable increase in CD4 lymphocyte counts and a reduction in plasma HIV‐RNA viral load, leading to a significant decrease in the prevalence of various oral lesions associated with HIV (de Brito et al. 2009; Greenspan et al. 2001). The impact of HAART has also been evident in the reduction of HIV‐related secondary events, such as opportunistic infections, including oral candidiasis and herpesvirus infections (Fontes et al. 2014; Xu et al. 2024).

In addition to improving the clinical status of HIV patients, HAART has substantially reduced AIDS‐related morbidity and mortality by promoting viral suppression and partial restoration of immune function. The therapy, consisting of more than 30 drugs across six distinct classes, has become the standard treatment for HIV infection (Nittayananta et al. 2010). Depending on the case, different classes of medications—such as nucleoside and non‐nucleoside reverse transcriptase inhibitors and protease inhibitors—are combined to maximise therapeutic efficacy and minimise viral resistance (Nittayananta et al. 2010; Hammer et al. 2008). With the advancement of HAART, there has been an improvement in the quality of life and prognosis for millions of individuals living with HIV worldwide (Nittayananta et al. 2010; Hammer et al. 2008; Lederman 2001).

Despite the remarkable advances in potent antiretroviral therapies in reducing HIV‐related mortality and morbidity, pain remains a frequently reported issue among HIV‐positive patients. It is estimated that the prevalence of chronic pain, regardless of body region, ranges from 25% to 80% in this population. This suggests that pain may be more common among HIV‐positive patients than in the general population, where rates vary from 20% to 30% (Sharma et al. 2018). Chronic pain in HIV‐positive individuals may have multiple causes, including the disease itself, side effects of treatments or associated chronic conditions (Aouizerat et al. 2010). Furthermore, the presence of psychiatric comorbidities and intravenous drug use is often observed in HIV patients, increasing the complexity of patient management (Xu et al. 2024; Sharma et al. 2018; Merlin et al. 2012).

Despite effective viral suppression achieved with HAART, people living with HIV continue to experience a meaningful symptom burden driven by persistent low‐grade inflammation and immune dysregulation (Wilson et al. 2016). As these individuals must manage the long‐term systemic consequences of HIV infection in parallel with the demands of continuous antiretroviral therapy (Günthard et al. 2016), any additional inflammatory stimulus—such as post‐operative pain following endodontic treatment—may have a different clinical impact. Accordingly, assessing post‐operative endodontic pain in patients receiving HAART is warranted to determine whether this population requires tailored or modified pain‐management strategies.

Some dental treatments are directly associated with post‐operative pain episodes, such as endodontic treatment (Iparraguirre et al. 2024). Post‐operative pain in endodontics is a recurring concern among patients undergoing root canal therapy, with prevalence ranging from 3% to 58% of cases (Ajeesh et al. 2023; Gundogdu and Arslan 2018; Ferreira et al. 2020). The intensity of this discomfort can vary from mild to severe and is caused by several factors, including mechanical, chemical, or microbiological trauma to the periapical tissues (Ferreira et al. 2020; Hespanhol et al. 2022). Additionally, the extrusion of irrigants beyond the apical foramen is a factor that may worsen inflammatory symptoms and cause pain (Gundogdu and Arslan 2018; Hespanhol et al. 2022).

There has been a growing number of studies evaluating the quality of life in patients living with HIV, as well as the impact of HAART in this regard (Brechtl et al. 2001). Considering the trend in the literature indicating a chronic pain prevalence of up to 80% in HIV‐positive patients (Nittayananta et al. 2010; Sharma et al. 2018), this study aims to compare the level of post‐operative pain in HIV‐negative and HIV‐positive patients undergoing HAART following endodontic treatment. The null hypothesis is that systemic condition (HIV‐positive under HAART) has no effect on post‐operative pain in patients with asymptomatic apical periodontitis.

## 2 Materials and Methods

This study was approved by the local ethics committee under approval number 3.227.397 and registered with the International Standard Randomised Controlled Trial (ISRCT N1693 9754). The study population was selected from a sample of patients with dental complaints who were referred by the Counselling and Guidance Center (COA) of Curitiba for evaluation at the dental clinic of the Pontifical Catholic University of Paraná.

A sample size calculation was performed using the G*Power 3.1 software (Heinrich‐Heine, Dusseldorf, Germany), with an alpha error of 0.05 and a beta power of 0.95. Sample size calculation estimated the desired size at 30 individuals per group. This research was determined by conducting a pilot study with 20 patients. The power effect size for a pilot study was 89, 80%. The sample was adjusted to account for approximately 10% of patients who may not respond to the VAS forms.

Inclusion criteria were: age ≥ 18 years; only single rooted teeth, meaning that anterior and premolar teeth were included in this study. Teeth had to be diagnosed with pulp necrosis and asymptomatic apical periodontitis, with digital radiographic evidence of apical periodontitis (minimum size 2 × 2 mm). For apical periodontitis diagnosis, the documented radiolucencies were also based on the apical periodontium status which was assessed by a 5‐score periapical index (PAI) described by Orstavik et al. (1986). PAI ≥ 4 was considered indicative of periapical lesion.

Pulp vitality was assessed before the beginning of the treatment using Endo Ice spray (Maquira, Maringá, Brazil). The pulp necrosis was confirmed using a combination of clinical tests and diagnostic findings, including cold test results that were performed by an endodontic specialist to ensure accuracy, crown condition (natural crown colour or full‐coverage restorations), and caries depth. However, in all cases in which the response to the cold test was doubtful or not sufficiently clear, an electric pulp test was also performed to confirm the diagnosis.

For the HIV+ group, additional criteria included: patients confirmed HIV‐positive for HIV antibodies through a particle agglutination test (SERODIA HIV, Fujirebio Inc., Shinjuku‐ku, Tokyo, Japan) and enzyme‐linked immunosorbent assay (ELISA) (Enzygnost Anti‐HIV ½ Plus, Behring, Behringwerke AG, Marburg, Germany), and on HAART for a minimum period of 3 years with an undetectable viral load.

Exclusion criteria included individuals with acute apical pain, those with chronic use of corticosteroids or analgesics, patients using antibiotics, systemic disorders, allergic reactions to dental materials, pregnancy, previous RCT, vital teeth, teeth with anatomical foramina (assessed during root canal therapy) with a diameter > #20 K‐type or < #10 K‐type instruments, and teeth with root resorption compromising the apical region. Only one tooth per patient was included in the study. All patients were informed about the study design and objectives and written informed consent was obtained before treatment. The sex, age, treated tooth and tooth location in the dental arches were recorded.

A total of 60 patients (30 HIV‐positive under HAART and 30 HIV‐negative) with pulp necrosis and asymptomatic apical periodontitis were included. All participants received a questionnaire based on the VAS to record their pain levels during treatment and at 6 h, 12 h, 24 h, 48 h, 72 h and 7 days post‐treatment; pain scores ranged from 0 to 10, with 0 representing no pain and 10 representing unbearable pain. The number of patients reporting pain levels between 0 and 10 from 6 h to 7 days was recorded. All recruited patients reported no orofacial pain prior to RCT and only patients with pre‐operative pain scores of 0 were included (Iparraguirre et al. 2024).

RCT was performed in a single visit by an endodontic specialist. Local anaesthesia was administered to ensure patient comfort, as rubber dam clamp placement and manipulation of periapical tissues can still cause discomfort even in non‐vital teeth. Patients were anaesthetised using 1.8 mL of 2% mepivacaine with epinephrine 1:100000 (DFL, Rio de Janeiro, Brazil) via buccal infiltration. Supplementary anaesthesia via palatal or lingual infiltration was administered when necessary. Access cavities were created using a high‐speed round diamond bur. Root canals were pre‐flared using a rotary SX instrument (17.08) (Dentsply Sirona, Ballaigues, Switzerland). The canals were explored and apical patency achieved using special series K‐files #06, 08 and 10 (Dentsply Sirona, Ballaigues, Switzerland). Working length (WL) was determined using an electronic apex locator (‘Root ZX II’; J. Morita, Tokyo, Japan) with the most appropriate K‐file for the canal anatomy. The WL was set 1 mm short of the 0.0 mark on the locator display. Root canal preparation was carried out using reciprocating mechanised instruments mounted on a Smart Plus motor (Dentsply Sirona, Ballaigues, Switzerland). The canals were irrigated with a total of 15 mL of 2.5% sodium hypochlorite (Asfer, São Caetano do Sul, Brazil), using 3 mL after each instrument change during canal exploration (files #6–10) and 4 mL after each instrument change during canal preparation with reciprocating instruments 25.08, 40.06, 50.05 (VDW, Munich, Germany). Positive‐pressure irrigation was performed between each instrument using a Max‐i‐Probe 30 G needle (Dentsply Sirona, Ballaigues, Switzerland), positioned 3 mm short of the working length as determined by a silicone stop. The irrigating solution remained inside the root canal throughout the procedure. Instrument debris was removed with gauze soaked in 70% alcohol after every three pecking motions.

A final irrigation was performed using 5 mL of 17% ethylenediaminetetraacetic acid (EDTA) (Biodinâmica, Ibiporã, Brazil), applied gently 1 mm short of the working length and left inside the canal for 1 min. All canals were dried with sterile paper points, then obturated using a single gutta‐percha cone matching the final instrument size (Reciproc, VDW, Germany) and AH Plus sealer (Dentsply Sirona, Ballaigues, Switzerland). The access cavity was temporarily sealed with Bulk Fill Flow composite resin (3 M ESPE, St. Paul, MN, USA). The quality of obturation was verified with a periapical radiograph to confirm that the established working length was maintained. In all cases treated in two groups, the occlusal reduction was performed after the treatment as a strategy for the prevention of post‐operative pain.

Patients were informed about the possibility of experiencing pain in the days following treatment and were given a take‐home questionnaire containing six VAS forms. They were instructed to record their pain levels at 6 h, 12 h, 24 h, 48 h, 72 h, and 7 days post‐treatment and were also contacted by phone at each interval as a reminder to complete the VAS data. Patients were also instructed to contact the researchers in case of severe pain, in which case 400 mg of ibuprofen would be prescribed and this would be recorded in the questionnaire (Iparraguirre et al. 2024). After 7 days, the patients returned for permanent restoration of the treated tooth using composite resin (3 M ESPE, St. Paul, MN, USA).

For statistical analysis, the chi‐square test was used to assess associations between gender, age and tooth location (Table 1). The Kolmogorov–Smirnov test was used to analyse data distribution. The test results showed that the pain level data were not normally distributed. Therefore, the non‐parametric Mann–Whitney *U* test was used for independent samples (Table 2). The confidence interval was set at 95% for all tests.

**TABLE 1:** Distribution of variables: Sex, age and tooth location between groups.

	HIV‐ *n* = 30	HIV+ *n* = 30
Age		
< 30	3	3
30–50	16	18
50 >	11	9
Location		
Mandibular	22	9
Maxillary	8	21
Sex		
Female	10	6
Male	20	24

**TABLE 2:** Median and interquartile range (IQR) of VAS pain scores according to the groups.

	HIV‐	HIV+	*p*
Initial	0.00 ± 0.00	0.00 ± 0.00	1
6 h	0.00 ± 2.00	1.00 ± 2.00	0.486
12 h	0.00 ± 1.00	1.00 ± 2.00	0.252
24 h	0.00 ± 0.00	0.00 ± 2.00	0.607
48 h	0.00 ± 0.00^a^	0.00 ± 0.00^b^	0.021*
72 h	0.00 ± 0.00	0.00 ± 0.00	1
7 days	0.00 ± 0.00	0.00 ± 0.00	1

*Note:* Different letters indicate statistically significant differences between the groups at the 48‐h interval (*p* < 0.05).

## 3 Results

The chi‐square test indicated that, regarding systemic condition in both groups, there was no association with sex, age or tooth location (*p* > 0.05) (Table 1).

Table 2 presents the descriptive differences (mean and standard deviation) in pre‐operative and post‐operative VAS pain scores according to the different groups and time intervals. A statistically significant difference was observed at the 48‐h time point (*p* = 0.021), where the HIV+ group showed complete pain relief, while the HIV– group still reported some level of pain. No patient in either group required rescue analgesics (400 mg ibuprofen) during the 72‐h post‐operative period.

## 4 Discussion

Pain in HIV‐positive patients has been the subject of study over recent decades due to the continuous evolution of HIV treatment and its potential impact on health‐related quality of life (Sharma et al. 2018; Aouizerat et al. 2010). Before the advent of HAART, pain was considered a common symptom among HIV‐positive patients (Aouizerat et al. 2010). This high prevalence of pain could be explained by multiple factors, such as the disease itself, chronic conditions, consequences of other treatments, underestimation of pain severity by healthcare providers or the fact that studies at the time focused on patients with AIDS or those in terminal stages (Aouizerat et al. 2010; Larue et al. 1997).

With the advent of HAART, research involving HIV‐positive patients entered a new era (Gonçalves et al. 2007). In dentistry, studies have shown that patients undergoing treatment may exhibit a reduced frequency of oral lesions (Greenspan et al. 2001), a lower diversity of bacterial species in chronic periodontitis cases (Gonçalves et al. 2007), and a decreased risk of orofacial pain (Nittayananta et al. 2010). In the field of endodontics, this is the first study to evaluate post‐operative pain levels following RCT in asymptomatic HIV‐positive patients. The inclusion of patients with pulp necrosis and asymptomatic apical periodontitis, the exclusion of those with pre‐operative pain and the seven‐day follow‐up period were selected to reduce confounding variables and to provide a clear assessment of pain exclusively related to the endodontic procedure.

A systematic review by Sathorn (Sathorn et al. 2008) demonstrated that post‐operative endodontic pain can occur in up to 58% of cases. Similarly, our study showed a prevalence of post‐operative pain in over 45% of cases, regardless of the patients' systemic condition. Additionally, factors such as over‐instrumentation beyond the apical foramen during chemo‐mechanical preparation may lead to an acute inflammatory response due to iatrogenic trauma in the periapical region (Yaylali et al. 2017; Machado et al. 2021). Consequently, obturation may result in increased extrusion of filling material, potentially provoking a severe inflammatory reaction in the periapical tissues (Iparraguirre et al. 2024). To minimise the risk of post‐operative pain, all cases in this study were treated 1 mm short of the 0.0 mark on the electronic apex locator. This highlights the clinical relevance of post‐operative pain as an outcome, since its occurrence directly affects patient comfort and perception of treatment success.

The null hypothesis in this study was rejected. Our results indicate that, after the first 48 h, all HIV‐positive patients reported complete pain relief, which was significantly different (*p* < 0.05) from the HIV‐negative group at the same time interval. Regardless of the patients' systemic condition, there was no significant difference in post‐operative pain during the first 24 h (*p* > 0.05). This initial finding reinforces what was previously reported by Nittayananta et al., (Nittayananta et al. 2010), who compared the oral health of HIV‐positive and HIV‐negative patients and found that both groups may present a similar risk of orofacial pain, regardless of systemic condition. Only after 72 h of treatment did both groups report complete pain relief.

Studies have shown that chronic pain is associated with various psychosocial factors, such as reduced health‐related quality of life, functional impairment and psychological distress (Sharma et al. 2018; Merlin et al. 2012). In Brazil, the national STD/AIDS programme provides counselling and universal access to antiretroviral therapy for HIV‐positive patients (de Brito et al. 2009). This support, which has expanded over the years, along with appropriate and continuous treatment, may have contributed to improved physical and emotional quality of life.

The HIV‐positive patients in this study did not report any chronic systemic pain conditions prior to endodontic treatment; therefore, only acute pain resulting from endodontic treatment was evaluated. Considering that the HIV‐positive participants in this study had been on consistent and continuous HAART for more than three years, it is possible that the absence of acute post‐operative pain within the first 48 h may be related to long‐term exposure to antiretroviral agents, which may alter pain thresholds when compared to individuals not taking such medications. A common side effect of HAART is peripheral neuropathic chronic pain (Addis et al. 2020). Although none of the patients in this study reported this condition, the possible side effects of HAART must be taken into consideration.

There is evidence that long‐term HAART exposure may alter nociceptive processing. Several antiretroviral agents—particularly nucleoside reverse transcriptase inhibitors—are associated with mitochondrial dysfunction, oxidative stress, inflammatory dysregulation and peripheral neurotoxicity, which can lead to neuropathic pain and hyperalgesia. Protease inhibitors may further potentiate these effects (Addis et al. 2020). Consequently, patients living with HIV may present modified pain thresholds or chronic neuropathic symptoms, potentially influencing the perception and reporting of acute post‐operative endodontic pain.

Most studies on HIV and endodontics have focused on healing and treatment success rates (Quesnell et al. 2005; Alley et al. 2008; Tootla and Owen 2012; de Araujo et al. 2024). However, there remains a gap in the literature regarding short‐term post‐operative pain. The strengths of the present study include the prospective observational design, standardised clinical protocol and the homogeneity of groups with respect to age, sex and tooth location. Nevertheless, some limitations must be acknowledged. The relatively small sample size and the absence of randomisation may reduce the power of the findings and residual confounding variables, such as individual pain sensitivity or psychosocial factors, may have influenced pain perception. Despite these limitations, the standardisation of procedures contributes to the internal validity of the study.

The generalisability of these results should be interpreted with caution. The findings may apply to HIV‐positive patients on long‐term HAART with undetectable viral load but cannot be extended to patients with advanced disease, poor adherence to therapy, or without regular medical follow‐up. Future research should involve larger and more diverse samples, evaluate additional systemic conditions, and investigate potential mechanisms by which antiretroviral therapy might influence pain perception.

## 5 Conclusion

Post‐operative pain following endodontic procedures exhibited similar patterns between groups, with complete pain relief observed after 48 h in the HIV‐positive group, suggesting that systemic condition does not influence the management of post‐operative endodontic pain.

## Author Contributions

**Everdan Carneiro:** conceptualization, methodology (lead), project administration, supervision, data analysis (equal), visualisation, writing – original draft preparation, review and editing (lead). **Marco Antonio Hungaro Duarte:** data analysis (equal), supervision, writing – original draft preparation. **Marcos Felipe Iparraguirre Nuñovero:** methodology, investigation, writing – original draft preparation, validation. **Ulisses Xavier da Silva Neto:** methodology, investigation, writing – review and editing. **Luciana Reis Azevedo Alanis:** investigation, writing – review and editing. **Bruno Cavalini Cavenago:** investigation, writing – review and editing.

## Funding

The authors have nothing to report.

## Conflicts of Interest

The authors declare no conflicts of interest.

## Data Availability

The data that support the findings of this study are available on request from the corresponding author. The data are not publicly available due to privacy or ethical restrictions.
